# Expression, Purification, and In Silico Characterization of *Mycobacterium smegmatis* Alternative Sigma Factor SigB

**DOI:** 10.1155/2022/7475704

**Published:** 2022-05-20

**Authors:** Rakesh Kumar Singh, Lav Kumar Jaiswal, Tanmayee Nayak, Ravindra Singh Rawat, Sanjit Kumar, Sachchida Nand Rai, Ankush Gupta

**Affiliations:** ^1^Molecular Microbiology Laboratory, Department of Biochemistry, Institute of Science, Banaras Hindu University, Varanasi-221005, Uttar Pradesh, India; ^2^Centre for Bioseparation Technology, Vellore Instiute of Technology, Vellore-632014, Tamil Nadu, India; ^3^Centre for Biotechnology, University of Allahabad, Prayagraj-211002, Uttar Pradesh, India

## Abstract

Sigma factor B (SigB), an alternative sigma factor (ASF), is very similar to primary sigma factor SigA (*σ*^70^) but dispensable for growth in both *Mycobacterium smegmatis* (Msmeg) and *Mycobacterium tuberculosis* (Mtb). It is involved in general stress responses including heat, oxidative, surface, starvation stress, and macrophage infections. Despite having an extremely short half-life, SigB tends to operate downstream of at least three stress-responsive extra cytoplasmic function (ECF) sigma factors (SigH, SigE, SigL) and SigF involved in multiple signaling pathways. There is very little information available regarding the regulation of SigB sigma factor and its interacting protein partners. Hence, we cloned the SigB gene into pET28a vector and optimized its expression in three different strains of *E. coli*, viz., (BL21 (DE3), C41 (DE3), and CodonPlus (DE3)). We also optimized several other parameters for the expression of recombinant SigB including IPTG concentration, temperature, and time duration. We achieved the maximum expression of SigB at 25°C in the soluble fraction of the cell which was purified by affinity chromatography using Ni-NTA and further confirmed by Western blotting. Further, structural characterization demonstrates the instability of SigB in comparison to SigA that is carried out using homology modeling and structure function relationship. We have done protein-protein docking of RNA polymerase (RNAP) of Msmeg and SigB. This effort provides a platform for pulldown assay, structural, and other studies with the recombinant protein to deduce the SigB interacting proteins, which might pave the way to study its signaling networks along with its regulation.

## 1. Introduction

Prokaryotic transcription is mediated by RNA polymerase (RNAP) which is made up of five core subunits (*α*_2_*ββ*′*ω*) that binds with the sigma factor (*σ*), also known as transcription initiation factor that provides specificity during transcription initiation. Based on the structure and function, *σ* factors are classified into two major groups, viz., (i) sigma 70 (*σ*^70^), i.e., primary sigma and (ii) sigma 54 (*σ*^54^) which is involved in nitrogen fixation and found mostly in plant growth promoting rhizobacteria. Further, *σ*^70^ is classified into four major groups based on the domain organization, viz., groups 1, 2, 3, and 4. Group 1 (SigA) has an extended N-terminal region and four highly conserved regions, namely, regions 1, 2, 3, and 4. The group 2 (SigB) does not have the N-terminus extended region, but all the four conserved regions are present. The group 3 (SigF) has only three regions, viz., 2, 3, and 4 while the group 4 (also known as the Extra Cytoplasmic Function (ECF) sigma factor) contains only regions 2 and 4. The regions 2 and 4 are extremely important in promoter recognition and binds at -10 and -35 regions of the promoter, respectively [1, 2].

*σ* ^70^ being the principle *σ* factor contributes in the expression of all housekeeping genes under normal conditions [3, 4]. However, under various stressful conditions, alternative sigma factors (ASF) redirect the transcription machinery towards the expression of a wide range of genes, which help the organism to respond to the internal or environmental stresses [5, 6]. Due to unequal division of tasks, *σ*^70^ has broad range promoter recognition efficiency but ASF deal with very high promoter recognition stringency. By these mechanisms that regulate transcription initiation, the organism ensures its survival and adaptability [7–9]. SigB responds to different stresses like heat shock, surface, oxidative, starvation stresses, and macrophage infections [3, 10, 11]. It is also expressed while transition to stationary phase during bacterial growth to cope with the environmental stresses [12–15].

In contrast to SigA, the half-life of Msmeg SigB is very short (less than 8 mins.) and in *E. coli*, it is only 2 minutes [12, 16–18]. This indicates towards the nature and role of SigB as a protein with a short half-life and involved in regulatory functions. The transcription of SigB was shown to occur from two distinct types of promoters: one, recognized by the stress-inducible sigma factors, viz., SigE, SigH, and SigL and the other recognized by SigF [19–22]. This infers that multiple sigma factor signaling networks converge at the SigB promoter. On the other hand, this observation is also supported by the deletion analysis of SigC, SigE, SigF, SigH, and SigL sigma factors resulting in the reduced expression of SigB, demonstrated in the mice model [19, 23–26]. The regulatory network and structure of the primary sigma factor SigA are already deduced. However, very little information about the regulatory network of SigB and other stress-responsive ECF sigma is available in the literature. Although recombinant and purified SigB is essential for the study of protein interactions/elucidation of its regulatory network as well as crystallization studies, detailed and optimized expression and purification of this short-lived protein in higher quantities and highly soluble form are not available in the literature. Here, we optimize and demonstrate the expression and purification of highly soluble Msmeg SigB.

## 2. Material and Methods

### 2.1. Bacterial Strains, Chemicals, and Reagents

*E.coli* DH5*α* (Zymo Research, USA) was used as cloning host, and BL21 (DE3), C41 (DE3) (Millipore Sigma (Novagen)), and CodonPlus (DE3) (Agilent Technologies) cells were used as expression host strains. The pET28a expression vector (Thermo Scientific, USA), Taq DNA polymerase (G-Biosciences, USA), restriction enzymes (New England Bio Labs Ltd., UK), T4 DNA ligase (New England Bio Labs Ltd., UK), and molecular biology grade reagents were obtained from Sigma-Aldrich (USA). His-Tag monoclonal antibody and secondary anti-mouse IgG (H + L) (peroxidase/HRP conjugated) were from Puregene: Genetix Biotech Asia Pvt. Ltd., respectively.

### 2.2. Plasmid Construction

The nondirectional cloning strategy was employed for the cloning of *SigB* (MSMEG_2752) gene of *M. smegmatis* mc^2^155. The primer pair used to amplify the *SigB* gene from the genomic DNA of *M. smegmatis* mc^2^155 was forward primer SigB_2752_EcoRI 5′-CGGAATTCATGGCAAATGCCACCACAAGCC-3′ and reverse primer SigB_2752_EcoRI 5′-CGGAATTCGGAGGCGTAGGAGCGGAGGCGG-3′ respectively, where underline sequences are showing the EcoRI restriction site. The condition for PCR amplification was initial denaturation at 95°C for 5 minutes, 35 cycles (denaturation 95°C for 30 sec, annealing 60°C for 45 sec, elongation 72°C for 1 minute), and final elongation 72°C for 5 minutes. The amplified PCR product was ligated into the EcoRI digested and dephosphorylated pET28a expression vector. The ligation product was transformed into DH5*α* cells, and the recombinant positive clones (His-SigB) were confirmed by EcoRI restriction digestion and sequencing.

### 2.3. The Expression of Recombinant His-SigB Protein

#### 2.3.1. Expression Host Strain Optimization

The clones containing His-SigB in positive orientation were transformed into three expression host strains of *E.coli*, viz., BL21 (DE3), C41 (DE3), and CodonPlus (DE3) [27]. These expression strains possess *λ*DE3 lysogen that carries the gene T7 RNA polymerase under the control of lacUV5 promoter. So, IPTG (isopropyl *β*-D-1-thiogalatopyranoside) induction for the protein expression was performed to obtain maximum expression. The selected clone was inoculated from a single colony and cultured overnight in 5 ml Luria Bertani (LB) containing 50 *μ*g/ml kanamycin at 37°C in a shaking incubator at 150 rpm. Next day, 1% (v/v) of secondary culture was incubated at 37°C until the OD_600_ of the culture reached ~0.4 O.D., and the culture was induced with 0.1 mM IPTG and grown for 3 hrs at 37°C at 150 rpm. For analysis of the His-SigB protein expression, sodium dodecyl sulphate polyacrylamide gel electrophoresis (SDS-PAGE) was performed as described by [28]. For SDS-PAGE, 0.1 OD_600_ equivalent cell lysate prepared in 4× sample loading buffer (10% SDS, 40% glycerol, 0.25 M Tris–Cl (pH 6.8), 200 mM DTT, and 0.05% bromophenol blue) after heat denaturation at 100°C was loaded onto the gel. For visualization, the gel was stained by Coomassie brilliant blue (R-250) followed by destaining.

#### 2.3.2. IPTG Optimization

Commonly, IPTG was used to achieve high level of expression for recombinant proteins. To optimize the concentration of IPTG on *E. coli* strains and the optimum protein expression, we selected best expressing host strain for a range of IPTG concentrations, viz., 0.05, 0.1, 0.25, and 0.5 mM and induced at approximately <0.4 OD_600_ at 37°C 150 rpm for 3 hrs. To determine the solubility of protein at various IPTG concentrations, the pellet/supernatant fractionation of the lysate was performed. The harvested cell cultures were washed and resuspended in 1× phosphate buffer saline (PBS, pH 8.0) and thereafter sonicated at 15% amplitude (ultrasonic homogenizer) for 2-3 min. The lysate was clarified by centrifugation at 13,000 rpm for 20 min at 4°C containing soluble His-SigB in the supernatant fraction while the pellet fraction was resuspended in equal volume of lysis buffer (1× PBS). The protein bands were visualized by electrophoresis in denaturing SDS-PAGE after staining with Coomassie R-250 as mentioned above. Semiquantitative analysis of the gels was performed with Quantity One software (Bio-Rad).

#### 2.3.3. Temperature Optimization

To optimize suitable temperature for protein expression, the best expressing host strain cultured at different temperatures, viz., 37°C, 25°C, and 16°C. The cells were induced with 0.1 mM IPTG at 37°C and 25°C for 3 hours, while at 16°C for 12 hours with shaking at 150 rpm. The protein bands were visualized by SDS-PAGE, and solubility fractionation analysis was done as above.

#### 2.3.4. Time Point Optimization

To check the best time point for the expression of His-SigB in the best expressing host strain, it was induced with 0.1 mM IPTG at 25°C/150 rpm for different time intervals, viz., 0, 15, 30, 60, 120, and 180 min. The expression profile was visualized in 10% SDS PAGE.

### 2.4. His-SigB Purification and Immunoblotting

The recombinant His-SigB was purified by affinity using Ni-NTA beads as per manufacturer's instructions. Briefly, the cells were lysed by sonication at 15% amplitude in buffer A (Tris.Cl 50 mM (pH 8.0), NaCl 300 mM, imidazole 10 mM, Triton X-100 1% (v/v), PMSF 2 mM, lysozyme 1 mg/ml), and the soluble lysate was clarified by centrifugation at 13,000 rpm (RCF 14,926 × g) for 15 min. at 4°C. The preequilibrated Ni-NTA beads (Qiagen) in buffer A were incubated with the lysate at 4°C for 30 mins., washed thrice with buffer B (Tris.Cl 50 mM (pH 8.0), NaCl 300 mM, Imidazole 50 mM, Triton X-100 0.5% (v/v)), and eluted thrice with buffer C (Tris.Cl 50 mM (pH 8.0), NaCl 300 mM, imidazole 400 mM, Triton X-100 0.5% (v/v)).

The purified His-SigB was resolved by SDS-PAGE and transferred to the PVDF membrane followed by blocking in 5% (w/v) skimmed milk powder (HiMedia laboratories) in 1× PBST (phosphate saline buffer containing 0.1% Tween-20). The blot was washed thrice by 1× PBST and incubated with anti-His antibody (0.5 *μ*g/*μ*l, 1 : 1000 in 1% PBST; Puregene) for 1 hr., washed with PBST, and further incubated with secondary antibody anti-Mouse IgG (H + L peroxidase/HRP conjugated, 1 : 5000 Pure gene Genetix Biotech) in PBST. Finally, the blot was washed thrice by PBST, developed in Clarity^Tm^ ECL Bio-Rad, and analyzed by the ChemiDoc Imaging System (Bio-Rad).

### 2.5. Homology Modeling and Structural Comparison of SigA and SigB

The protein sequences of *M. smegmatis* sigma factors SigA and SigB were retrieved from the National Center for Biotechnology Information (NCBI) database [29]. To construct the homology models, the amino acid sequences of both the proteins were submitted to Phyre^2^ server [30]. Phyre^2^ server utilizes multiple templates to produce the accurate model of the protein. In case of *M. smegmatis* SigA, three templates were used, namely, 5TW1 (RNA polymerase sigma factor) [31], 6C05 (crystal structure of RNA polymerase sigma factor from *M. tuberculosis*) [32], and 4YG2 (X-ray crystal structure of sigma 70 holoenzyme from *E. coli*) [33] with percent identities of 99%, 97%, and 55%, respectively. Similarly, the *M. smegmatis* SigB model was build using two templates 5TW1 (RNA polymerase sigma factor) [31] and 6C05 (crystal structure of RNA polymerase sigma factor from *M. tuberculosis*) [32] with percent identities of 65% and 64%, respectively. Both the models were validated on SAVES server using PROCHECK tool [34].

### 2.6. Protein-Protein Docking of Core RNA Polymerase (RNAP) and SigB

The protein-protein docking utilizes a multistage method for generating poses followed by their rankings. Here, we have used MOE platform [35] for the protein-protein docking experiment of core RNA polymerase (RNAP) and SigB upon importing RNAP (PDB ID: 6EYD [36]) to MOE (https://www.chemcomp.com/Products.htm), workbench. Structure issues such as missing atoms and missing chains were automatically corrected using quick prep module. Optimal hydrogen positions and charges were optimized using protonated 3D module using the MOE software recommended settings. We have used here core enzyme of 6YED [36] PDB without sigma factor A (SigA). Before commencement of docking, bad crystallographic contacts or other imperfect geometries were overcome by the energy minimization method using Amber EHT 10 force field recommended by the MOE platform. Energy minimize application itself can be used to adjust hydrogens and lone pairs and to calculate partial charges. Similarly, SigB protein was prepared before docking. The SigB model was considered as ligand, and PDB ID: 6YED [36] was considered as a receptor. After the docking run, the appropriate protein-protein fingerprints were generated for each pose which can be viewed using the PLIF visualization panel. Approximately, 100 coordinates were developed according to the binding energy. The best twenty dock complexes were considered for analysis.

## 3. Results

### 3.1. Expression Host Strain Optimization for the Expression of Recombinant His-SigB

**Figure 1:**
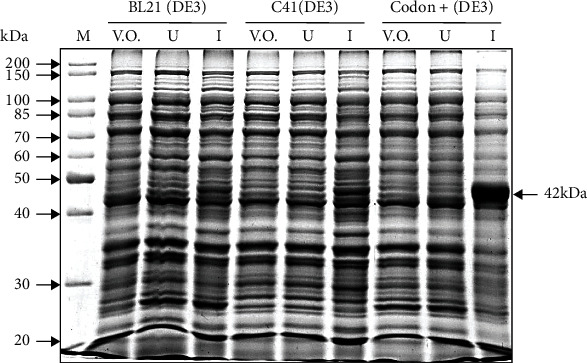
Expression host strain optimization for the expression of recombinant His-SigB. 10% SDS PAGE depicting recombinant His-SigB protein expression profile from three expression host strains, namely, BL21(DE3), C41 (DE3), and CodonPlus (DE3) expressed with 0.1 mM IPTG grown for 3 hrs at 37°C with shaking at 150 rpm. The highest protein expression is observed in the host strain CodonPlus (DE3). M: molecular size marker; V.O.: pET28a vector only control; U: uninduced His-SigB clone; I: induced His-SigB clone.

The pET28a vector belongs to the pET series of vectors that contain a T7 promoter with *lac* operator for the IPTG inducible expression of downstream recombinant genes. However, these genes can only be expressed when T7 RNA polymerase is made available in expression hosts containing a DE3 lysogen under the control of IPTG inducible lacUV5 promoter in their genomic DNA [27]. Hence, we utilized three expression host strains of *E. coli* containing the DE3 lysogen, viz., BL21 (DE3), C41 (DE3), and CodonPlus (DE3) for the optimization of the expression of recombinant His-SigB. Empty pET28a vector and uninduced recombinant His-SigB clones were used as negative control for the expression host strains BL21 (DE3), C41 (DE3), and CodonPlus (DE3) in Figure 1 (lanes: V.O. and U), respectively. These negative controls did not show any expression of the recombinant His-SigB as depicted in Figure 1. However, the maximum expression of the recombinant His-SigB (~42 kDa) is observed in the expression host CodonPlus (DE3) followed by C41 (DE3) and BL21 (DE3), respectively (lane I for each cell type in Figure 1).

### 3.2. Optimization of IPTG Concentration for Recombinant His-SigB

**Figure 2:**
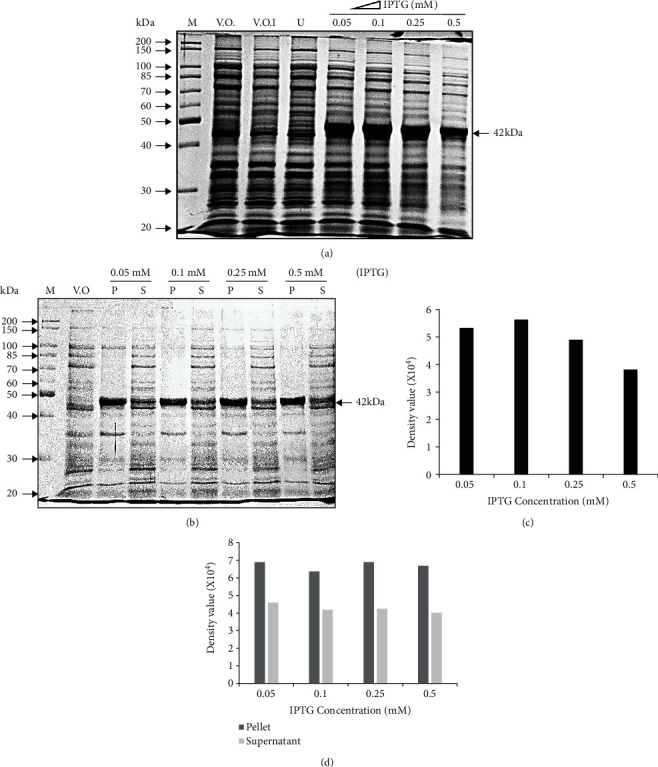
IPTG concentration optimization for the expression of recombinant His-SigB. (a) 10% SDS PAGE depicting recombinant His-SigB protein expression profile in CodonPlus (DE3) host strain. The highest protein expression is observed in 0.1 mM IPTG. (b) Solubility of the recombinant His-SigB in different IPTG concentrations at 37°C. 10% SDS PAGE depicting recombinant His-SigB protein distribution in cell pellet and supernatant in CodonPlus (DE3) induced with IPTG concentration from 0.05 to 0.5 mM IPTG. The highest protein solubility observed in the supernatant fraction at 0.1 mM IPTG. M: molecular size marker; V.O.: pET28a vector only control; V.O.I: pET28a vector only induced; U: uninduced His-SigB clone; P: pellet; S: supernatant. (c) Gel-based semiquantitative analysis from (a) also depicts the highest expression of His-SigB at 0.1 mM IPTG concentration. (d) Semiquantitative analysis from (b) depicts higher relative protein solubility observed in the supernatant fraction at 0.1 mM IPTG. Semiquantitative analysis of the gels was performed with Quantity One software (BIO-RAD).

CodonPlus (DE3) depicted the maximum expression of the recombinant His-SigB protein; hence, it was decided to optimize the maximum protein expression at different IPTG concentrations (0.05-0.5 mM). The expression of His-SigB at IPTG concentrations: 0.05, 0.1, and 0.25 mM was comparable which was approximately 30% higher than the expression at 0.5 mM concentration (as depicted in Figures 2(a) and 2(c)). Amongst the three concentrations, the best expression was found in 0.1 mM IPTG. When the pellet and supernatant fractions of the induced CodonPlus (DE3) cells were observed, comparable distribution of the soluble His-SigB protein was observed in the supernatant fractions at different IPTG concentrations as depicted in Figures 2(b) and 2(d). However, total expression and relative solubility of the recombinant His-SigB were optimum at 0.1 mM IPTG concentration which was selected further studies.

### 3.3. Temperature Optimization for the Expression of Recombinant His-SigB Protein

**Figure 3:**
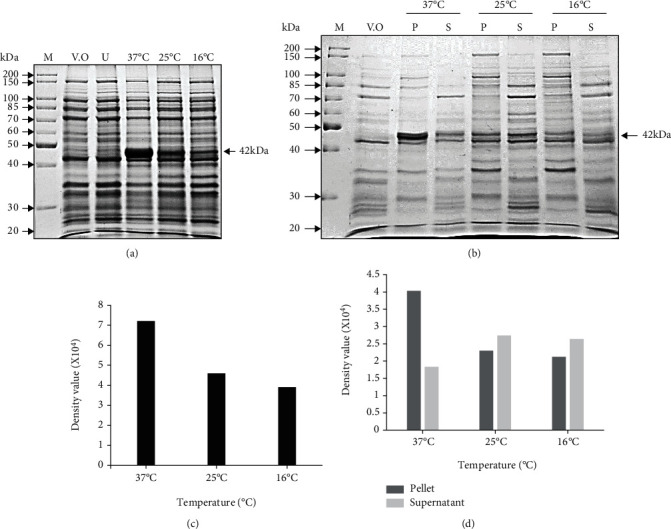
Temperature optimization for the expression of recombinant His-SigB protein. (a) 10% SDS PAGE depicting recombinant His-SigB protein expression profile in CodonPlus (DE3) cells at temperatures: 37°C, 25°C, and 16°C with 0.1 mM IPTG. The highest protein expression was observed at 37°C. (b) 10% SDS PAGE depicting distribution of recombinant His-SigB protein in cell pellet and supernatant fractions at different temperatures. The highest protein solubility was observed at 25°C supernatant fraction. M: marker; V.O.: pET28a vector only control; U: uninduced His-SigB clone; P: pellet; S: supernatant. (c) Gel-based semiquantitative analysis from (a) depicts the highest expression of His-SigB at 37°C with 0.1 mM IPTG concentration. (d) Semiquantitative analysis from (b) depicts higher relative protein solubility observed in the supernatant fraction at 25°C with 0.1 mM IPTG. Semiquantitative analysis of the gels was performed with Quantity One software (BIO-RAD).

At the optimized IPTG concentration of 0.1 mM, His-SigB was induced at 37°C as well as lower temperatures, viz., 25°C and 16°C for increasing the relative solubility of His-SigB. The maximum expression of His-SigB was obtained at 37°C and the expression decreased drastically to approximately 62% and 54% at temperatures: 25°C and 16°C, respectively (Figures 3(a) and 3(c) as compared to that at 37°C. However, when the distribution of the soluble His-SigB protein was observed in the supernatant fractions at different temperatures, it was observed that almost 31%, 55%, and 54% of the total induced proteins fractionated to supernatant at temperatures: 37°C, 25°C, and 16°C, respectively (Figure 3(b) and 3(d)). Thus, higher total expression and maximum relative solubility of the recombinant His-SigB were observed at 25°C temperature.

### 3.4. Time Course Experiments, Ni-NTA Affinity Purification, and Immunoblotting of Purified His-SigB

**Figure 4:**
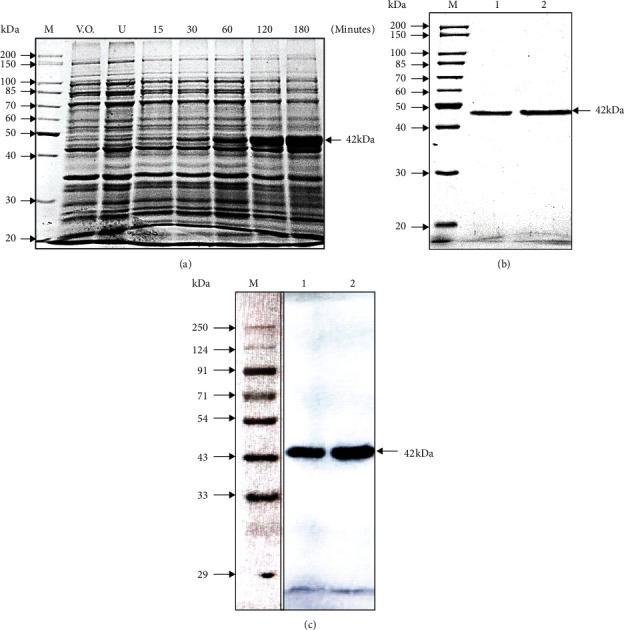
(a) The expression of recombinant His-SigB protein at different time points. 10% SDS PAGE depicting recombinant His-SigB protein expression profile collected at time points 15, 30, 60, 120, and 180 minutes. The highest protein expression was observed at 180 minutes after induction with 0.1 mM IPTG. (b) Ni-NTA affinity purification of His-SigB: lanes denoting M: molecular size marker; 1: 5 *μ*g; 2: 7.5 *μ*g His-SigB purified protein stained with Coomassie R-250. (c) Western blotting with purified recombinant His-SigB. Mouse anti-His monoclonal antibody (Genetix) was used to probe purified His-SigB. M: marker; V.O.: pET28a vector only control; U: uninduced His-SigB clone; lane 1: 5 *μ*g; lane 2: 10 *μ*g purified His-SigB protein.

The time course experiment was carried out with for determining the maximum expression of soluble His-SigB protein at the optimized conditions of 25°C with 0.1 mM IPTG for different time points, viz., 0, 15, 30, 60, 120, and 180 minutes. The maximum expression was observed at 180 minutes after the IPTG induction as depicted in Figure 4(a).

His-SigB protein was purified using Ni-NTA affinity chromatography. The affinity purified His-SigB protein was found to have minimal proteolytic degradation as depicted by the Coomassie stained gel in Figure 4(b). In spite of the short half-life of His-SigB, approximately 2 mg-2.2 mg/l culture of the protein was purified. To further rule out the possibility of proteolytic degradation of the recombinant protein, immunoblotting was performed using a monoclonal anti-6XHis antibody. It was observed that a highly purified His-SigB protein without any proteolytic degradation was obtained under the conditions optimized in this article as depicted in Figure 4(c).

### 3.5. Structural Characterization of SigA and SigB

The Ramachandran plots for *M. smegmatis* SigA and SigB proteins demonstrate 98.1% and 98.7% residues falling in the allowed regions, respectively (Supplementary Figure 1). The models of SigA and SigB, however, also exhibited few structural differences. *M. smegmatis* SigA differs from SigB in possessing an N-terminal extended domain. It has been observed that the presence of N-terminal extended domain provides stability to the molecule and consequently, and SigA is comparatively more stable than SigB.

**Figure 5:**
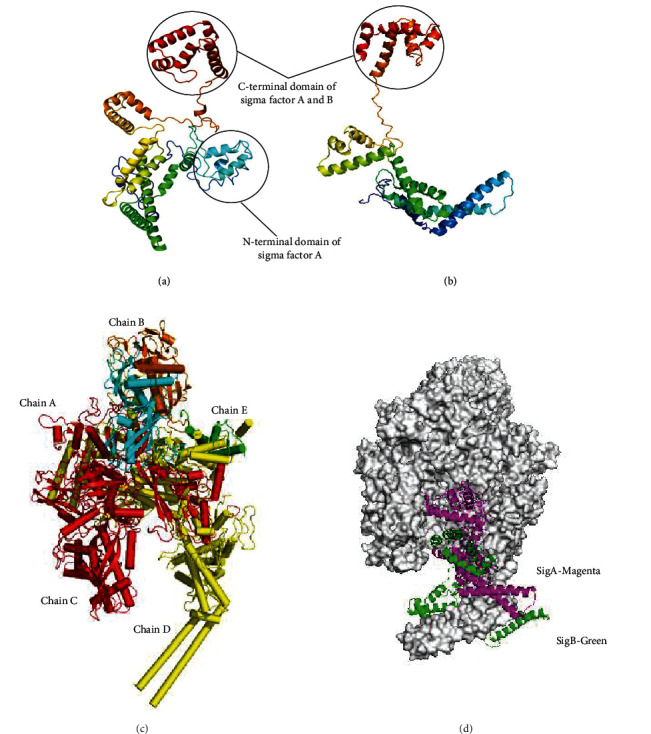
Homology models of (a) *M. smegmatis* sigma factor SigA, (b) *M. smegmatis* sigma factor SigB, (c) structure of the core RNA polymerase of *M. smegmatis*, and (d) comparative surface topology of SigA and SigB interactions with core RNA polymerase of *M. smegmatis.*

The C-terminal domains of SigA and SigB are composed of four *α*-helices. The first and the fourth *α*-helices of the domain lie adjacent to one another in a parallel fashion and run in opposite directions. The second and fourth helices are shorter than the first and third helices. Furthermore, second and fourth helices are connected to the ends of third helix in perpendicular manner through short loops (Figures 5(a) and 5(b)). The region connecting the first and second helix could not be modeled precisely.

### 3.6. Molecular Interactions of RNAP and SigB

Conformational changes of RNAP are required to initiate the transcription process. To investigate the interactions of SigB with RNAP, protein-protein docking experiment was performed. The protein-protein docking experiment revealed SigB bound to RNAP with binding energy of -70.39 (sixteen dock complexes). The *M. smegmatis* RNAP is composed of five subunits including *α* (two copies of chains A and B), *β* (chain C), *β*′ (chain D), and *ω* (chain E) (Figure 5(c). These five chains together formed the core enzyme and help to synthesize the RNA using DNA as template and ribonucleotides as the substrate. To start this process, the core enzyme has to bind to a sigma factor. In RNAP of *M. smegmatis*, chains C and D mainly interact with SigB (Figure 5(d)). Arg846 and Leu408 of chain C and Arg67, Thr38, Lys64, Thr150, Arg345, Glu250, and Glu275 of chain D play a major role in the molecular interactions with SigB which are in contrast with the molecular interactions of SigA.

## 4. Discussion

Although, SigB is dispensable for growth in both Msmeg and Mtb [15, 37], nevertheless, SigB acts as a general stress responsive transcription factor. The importance of SigB protein is elucidated by the fact that it occurs downstream in the signaling pathways of several stress responsive ECF and other sigma factors [13, 14, 22]. The expression of recombinant SigB has been mentioned in reference to the in vitro transcription assay experiment in BL21 (DE3) strain which is deficient of lon and omp-t proteases and is therefore suitable for expression of nontoxic genes but the SigB protein is very poorly expressed in this strain as demonstrated from our results (Figure 1) [14]. This leads us to express SigB in other alternative strains like C41 (DE3) and CodonPlus (DE3). C41 (DE3), derived from BL21 (DE3), contains an uncharacterized mutation that prevents cell death due to the expression of many toxic recombinant proteins. Hence, it is often utilized for the expression of toxic and membrane proteins from all classes of organisms [38]. However, in our case, it leads to only a marginal increase in the expression of recombinant SigB (Figure 1). CodonPlus (DE3) improves the protein expression by preventing codon bias and by providing additional copies of specific tRNA genes, viz., argU, ileY, leuW, and proL genes that recognize AGA/AGG, AUA, CUA, and CCC codons, respectively, that are rare in *E. coli* and occur commonly in GC-rich genomes like Mycobacterium [39, 40]. SigB of Msmeg in our case contains at least 8 prolines out of which 2 are encoded by CCC codons which might be an important reason for the lower expression in other strains utilized.

During the optimization with different IPTG concentrations, it was clearly observed that the total protein expression was higher at 0.1 mM IPTG concentration (Figures 2(a) and 2(c) with comparable relative expression of soluble SigB at IPTG concentrations like 0.05-0.25 mM (Figures 2(b) and 2(d). Since, the total expression was higher at 0.1 mM with comparable solubility; hence, 0.1 mM was finally selected for final expressions. Greater IPTG often leads to faster protein expression resulting in increased formation of inclusion bodies; hence, higher IPTG concentrations were not included for final expressions. Similarly, when three different temperatures were utilized for the SigB expression, there was a drastic decrease in the expression from 38% to 46% at 25°C and 16°C, respectively, as compared to that at 37°C. However, the relative fraction of total protein expressed as soluble fraction in the supernatant was much greater at lower temperatures like 25°C and 16°C. The intermediate temperature of 25°C was best suited for our experimental work (Figure 3). The IPTG induction of recombinant proteins like human RNase L, human growth hormone (hGH), and several other proteins at lower temperatures in bacteria favors relatively higher yield of the expressed protein in the soluble fraction of the cell [41–47]. The experimental conditions optimized above for the recombinant SigB expression provided a high yield of soluble and proteolytically undergraded protein which can be visualized by the immunoblotting of purified SigB using a monoclonal anti-6XHis antibody, since no degradation products could be visualized at higher concentrations of the purified protein (Figure 5).

However, the highly unstable nature of this protein can be further improved by using various stabilizers and (or) another cofactor so that future experiments like protein-protein interactions and crystallization of SigB can be carried out smoothly. *M. smegmatis* RNA polymerases SigA and Sig B showed 60% sequence identity. Sigma factor A differs from sigma factor B in possessing N-terminal domain, which may provide stability. Our docking studies demonstrated that SigB binds to RNAP where chain C and chain D of RNAP play a significant role in molecular interactions. In silico comparative binding studies of SigA and SigB demonstrated that they both bind to similar regions of RNAP. In future, our aim will be to cocrystallize SigB-RNAP together to better understand the mechanism of their molecular interactions.

## 5. Conclusion

SigB has significant similarities with house-keeping sigma factor SigA with regards to the domain organization; however, it has drastically short half-life as compared to SigA. Despite its short half-life, it is also involved in multiple stress response regulatory pathways of *Mycobacterium* in coordination with ECF and other sigma factors. Higher quantities of soluble and purified recombinant SigB are essential for the study of its structure, function, and regulation but there is dearth of literature that depicts it. In this report, we demonstrate an efficient method for the production of soluble and purified recombinant His-SigB protein that can be used as a tool to unravel the structure, function, and regulation of its signaling cascade.

## Data Availability

The data used in this study to support the findings are available from the corresponding author upon request.

## Supplementary Materials

Supplementary Materials Supplementary Figure 1: Ramachandran plots for the homology models of (a) *M. smegmatis* SigA and (b) *M. smegmatis* SigB.
