# Supplementary figures and images for: Expression, Purification, and In Silico Characterization of Mycobacterium smegmatis Alternative Sigma Factor SigB
Source: Dis Markers. 2022 May 20;2022:7475704. doi: 10.1155/2022/7475704 (PMC9142298; doi:10.1155/2022/7475704)

**Sup. fig. 1:** Ramachandran plots for the homology models of **a)** *M. smegmatis* SigA and **b)** *M. smegmatis* SigB

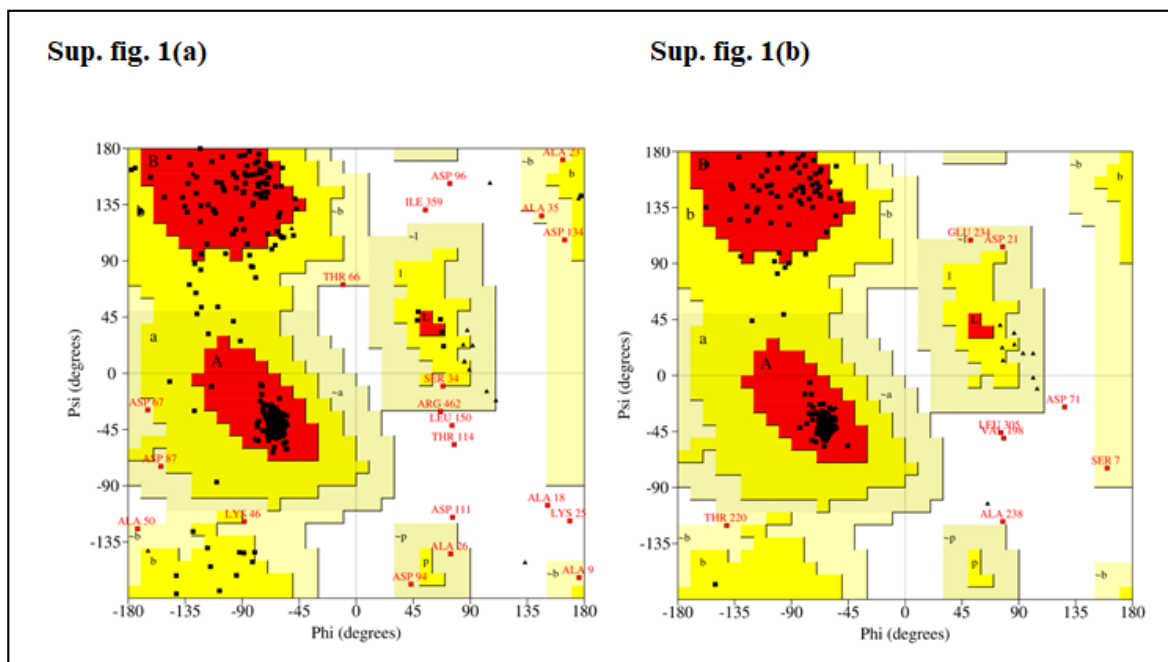

Supplement: Supplementary Materials — Supplementary Figure 1: Ramachandran plots for the homology models of (a) M. smegmatis SigA and (b) M. smegmatis SigB. [file 7475704.f1.pdf]
